# Differences in alexithymia, emotional awareness, and facial emotion recognition under conditions of self-focused attention among women with high and low eating disorder symptoms: a 2 x 2 experimental study

**DOI:** 10.1186/s40337-020-00304-5

**Published:** 2020-06-22

**Authors:** Jillon S. Vander Wal, Alicia A. Kauffman, Zachary A. Soulliard

**Affiliations:** 1 Department of Psychology, Saint Louis University, 3700 Lindell Blvd, St. Louis, MO 63108 USA; 2 Behavioral Medicine & Psychiatry, Chestnut Ridge Center, West Virginia University School of Medicine, 930 Chestnut Ridge Road, Morgantown, WV 26505 USA; 3 East Hawaii Family Guidance Center in Hilo, 88 Kanoelehua Avenue, Hilo, HI 96720 USA

**Keywords:** Eating disorders, Alexithymia, Theory of mind, Affect, Emotional regulation

## Abstract

**Background:**

Women with eating disorders generally perform more poorly on measures of alexithymia, defined as difficulty identifying and describing emotions, and theory of mind, or the ability to infer what others are thinking and feeling. The extent to which these abilities may be influenced by variables such as self-focused attention, or directing attention toward internally generated information, has yet to be investigated. Thus, the purpose of the present study was to examine differences between women high and low in disordered eating symptoms on measures of emotional awareness and facial affect recognition under conditions of high and low self-focused attention.

**Methods:**

University women scoring high or low on a measure of disordered eating (*n* = 79) were randomly assigned to a condition of high or low self-focused attention. Outcomes included alexithymia (self-rated ability to identify and describe emotions), emotional awareness (ability to describe the emotions of oneself and others), and facial affect recognition. Scores on a measure of negative affect were statistically controlled.

**Results:**

Women with high disordered eating symptom scores rated themselves as having more difficulties identifying, but not describing emotions after controlling for negative affect, but demonstrated greater difficulties describing their own and others’ emotions on a measure of emotional awareness. In the self-focused attention condition, women scored lower on self emotional awareness and were quicker to identify expressions of negative facial affect regardless of eating disorder symptom status than women in the non-self-focused attention condition. There were no significant interactions between eating disorder status and self-focused attention.

**Conclusions:**

Further examination of different types of emotion recognition and description in oneself and others as well as processes that may influence these abilities is warranted.

## Plain English summary

Women with eating disorders may find it harder to identify and describe emotions than women without eating disorders. These problems may be worse when women become focused on their own internal experiences (e.g., thoughts, memories, physical states). Participants included 79 university women who scored high (*n* = 40; high) or low (*n* = 39; low) on a measure of disordered eating. Half of each group, with their knowledge, was video-recorded to increase their focus on internal experiences. High scoring women rated themselves as having more problems identifying their emotions on a self-report inventory than low scoring women. High scoring women had greater difficulties identifying how they and someone else would feel when presented with a series of social scenarios than low scoring women. There were no differences between high and low scoring women on identifying facial expressions of emotion. Regardless of disordered eating score, women who were video-recorded had greater difficulties describing how they would feel in a social scenario and were quicker at identifying facial expressions of negative emotions than women who weren’t video-recorded. Results suggest the importance of using real-life scenarios when assessing these abilities, attending to negative emotions, and assessing the tendency to focus on internal experiences.

## Background

Alexithymia is defined as difficulty identifying and describing emotions, difficulty differentiating between emotional and physical cues, a restricted capacity for imagination, and an externally oriented thinking style [1]. Women with eating disorders score more poorly than healthy controls on self-report measures of alexithymia that assess difficulties identifying and describing emotions [2–6], although the results are weaker for women with bulimia nervosa than for women with anorexia nervosa [3, 5]. It is uncertain whether these deficits reflect the presence of alexithymia or an underlying mood disorder as differences between women with eating disorders and healthy controls are often diminished after controlling for anxiety and depression [4, 5, 7, 8], but not always [6]. However, difficulty identifying feelings is predictive of unfavorable eating disorder treatment outcomes, independent of initial symptom severity and depression [9]. Although alexithymia may diminish following recovery from an eating disorder, it tends to persist [2, 5, 10]. Finally, difficulties with alexithymia have also been found among non-clinical eating disordered samples [5].

Research has also examined emotional awareness, or the ability to infer emotional states in oneself and others from social scenarios among persons with anorexia nervosa. While over-lapping with the construct of alexithymia, this ability has also been characterized as a component of emotional theory of mind, or the ability to make inferences about another person’s feelings [10]. Reviews of this literature generally show impairments among persons with anorexia and bulimia nervosa [3, 10]. Four studies used a measure called the Levels of Emotional Awareness Scale [11, 12]. Participants were asked to read a social interaction vignette involving themselves and another person and write how they and the other person would feel. Participants showed impairments in the ability to describe how they and the other person would feel in three of the four studies [7, 8, 13]. No difference was found in the one study to employ a sub-clinical eating disordered sample [14].

While research supports the idea that women with eating disorders generally have difficulties identifying and describing emotions, other research has examined another aspect of emotional theory of mind—the ability to recognize facial emotional expressions. Reviews of this literature suggest that facial emotion recognition is impaired among persons with anorexia nervosa in comparison to healthy controls [3, 10]; however, both reviews note that several studies did not show a difference. Studies that employ more difficult facial emotion recognition tasks such as free-naming versus forced-choice emotion labels, presentation of mixed emotional states, or shorter exposure latencies tend to be more sensitive to these deficits [3, 10]. Women with bulimia nervosa generally do not show “substantial” impairments compared with healthy controls [3]. The authors caution that the limited number of studies precludes firm conclusions. A meta-analysis gave an overall effect size of *d* = .26 for facial emotion recognition across eating disorder diagnostic groups in comparison to healthy controls [2]; however, a Forest plot showed that this effect was driven by studies conducted among anorexia nervosa and mixed diagnostic eating disorder samples.

A variable that may influence emotional awareness and facial emotion recognition is self-focused attention (SFA). SFA has been defined as, “an awareness of self-referent, internally generated information that stands in contrast to an awareness of externally generated information derived through sensory receptors” [15] (p. 156). SFA is associated with a variety of clinical disorders and may serve as a vulnerability factor for psychopathology via intensification of salient internal mechanisms or as a cognitive diathesis [15]. Different models of the relationship between SFA and psychopathology have been proposed. According to Ingram’s meta-construct model [15], attentional shifts toward excessive, sustained, and inflexible SFA are thought to characterize self-absorption or a general vulnerability to psychopathology [15] while the specific content of SFA defines the form of psychopathology. Further, it is theorized that attention can be flexibly divided between one or more internal and/or external events simultaneously within one’s capacity. However, as attention to internal events increases, attention to external events decreases and vice versa.

Despite the strong association between SFA and psychopathology [15, 16], remarkably little work has been done on the association between SFA and disordered eating. It has been observed that the symptoms of anorexia nervosa, including the preoccupation with weight and shape, is indicative of narrowed SFA [17, 18]. One study found that an indicator of SFA (i.e., first-person singular pronoun use), was associated with symptoms of depression and anxiety during negative, but not positive memory recall among women with anorexia nervosa [17]. Another study found that increased SFA predicted disordered eating and body dissatisfaction among a group of college-aged female participants; furthermore, SFA partially mediated the relationship between body dissatisfaction and disordered eating in this study, with increased body dissatisfaction associated with increased SFA, which in turn was associated with increased eating disorder symptoms [19].

In apparent contrast to these findings, one study found that SFA was lowest among women with current anorexia nervosa compared with recovered women; healthy controls scored between these two groups [18]. Additionally, low levels of SFA were correlated with high levels of shape concerns particularly among women with anorexia nervosa [18]. The authors reasoned that the focus on weight, shape, and appearance among women with current anorexia nervosa distracted from SFA and was therefore negatively reinforcing. Their explanation brings these results into potential consistency with the meta-construct model as SFA appears to function in a similar capacity among persons with alcohol use disorders such that the consumption of alcohol inhibits SFA [15].

Finally, while SFA has not been experimentally induced in studies involving disordered eating, it has been induced in studies of depression and anxiety [15, 16]. As might be expected from the meta-construct model, persons with psychopathology may be more sensitive to such inductions than healthy controls. Indeed, an experimental manipulation to increase SFA did so more acutely among high versus low socially anxious participants [20].

In summary, research shows strong evidence for differences in self-reported alexithymia between women with eating disorders, particularly anorexia nervosa, and healthy controls, although these differences are often reduced after controlling for negative affect [4, 5, 7, 8]. Evidence also shows impairment in the ability to infer emotional states from social scenarios between women with eating disorders and healthy controls which tends to persist after controlling for negative affect [7, 8]. Evidence for impaired facial emotion recognition is stronger among samples with anorexia nervosa and weaker among samples with bulimia nervosa [3]. According to the meta-construct model [15], one would expect that as SFA increases, the ability to attend to external events, such as the emotional states of others, would decrease. Further, given that experimentally induced SFA intensifies psychopathology [15], one would expect a manipulation to increase SFA to do so more acutely among women with disordered eating symptoms than among healthy controls.

Thus, the purpose of the present study was to examine differences between women scoring high and low in disordered eating symptoms (High-EAT and Low-EAT respectively) on self-reported alexithymia, emotional awareness, and facial emotion recognition while manipulating SFA using a 2 × 2 experimental design. It was hypothesized that a) High-EAT women, in contrast to Low-EAT women, would endorse greater symptoms of alexithymia on a self-report measure of alexithymia; b) High-EAT women, in contrast to Low-EAT women, would score more poorly on measures of emotional awareness and facial affect recognition; c) women would score more poorly on measures of emotional awareness and facial affect recognition under conditions of SFA in comparison to no SFA (n-SFA); and d) SFA would impede performance to a greater extent among High-EAT than Low-EAT women (i.e., a significant interaction effect).

## Methods

### Aim

The aim of the study was to examine differences between High-EAT and Low-EAT women on self-reported alexithymia, emotional awareness, and facial emotion recognition while manipulating SFA using a 2 × 2 experimental design.

### Participants

The study was approved by the university’s Institutional Review Board charged with ensuring that research involving human subjects is conducted in accordance with the regulations, laws, and policies in place to protect the rights and welfare of human research volunteers. Participants were 79 women (*M* age = 18.58, *SD* = 0.87; *M* BMI = 22.85; *SD* = 3.31; range 16.75–36.23) from a Midwestern university in the United States who identified as being born in the United States or Canada, without visual impairments (defined as precluding them from driving), or upper body physical impairments (defined as conditions that would impact keyboarding speed). An eightieth woman was originally included, but later found to be ineligible. Participants were generally first year college students (74.7%), Caucasian/White (82.3%), and non-Hispanic (97.5%). Participants were separated into groups based on their scores on the Eating Attitudes Test-26 (EAT-26) [21]. We used a cut-score of 19 to identify women with high levels of disordered eating symptoms (High-EAT) and a score of 3 or less to identify women with low levels of disordered eating symptoms (Low-EAT) following the methods of previous studies [22]. Thus, thirty-nine women screened free of eating disorder pathology (*M* EAT-26 score = 1.69; *SD* = 1.08), while 40 women screened positive for eating disorder pathology (*M* EAT-26 score = 28.13; *SD* = 10.06), with 36 meeting or exceeding the cut-point of 20 thought to be indicative of an eating disorder [21].

### Screening measures

#### Demographics

A brief demographic form was used to ensure that participants met eligibility criteria.

#### The eating attitudes test--26 [21]

The EAT-26 is a 26-item measure of the thoughts, feelings, and behaviors associated with disordered eating. Higher scores indicate greater degrees of disordered eating [21]. Items are rated on a 6-point scale ranging from *never* to *always*. Response options of *never*, *rarely*, and *sometimes* receive scores of zero, whereas response options of *often*, *usually*, and *always* receive scores of 1, 2, and 3 respectively. The EAT-26 evidenced strong internal consistency reliability in the present study (*α* = .93).

### Study measures

#### The Toronto alexithymia scale—20 (TAS-20) [23]

The TAS-20 is a 20-item scale used to measure alexithymia. Items are rated on a 5-point scale, ranging from 1 (*strongly disagree*) to 5 (*strongly agree*), with higher scores indicating higher levels of alexithymia. Scores from 52 to 60 indicate possible alexithymia and scores of 61 or greater indicate probable alexithymia. The TAS-20 includes three factors including the ability to identify feelings and distinguish them from physical sensations of arousal (i.e., difficulty identifying feelings), the inability to communicate feelings to others (i.e., difficulty describing feelings), and externally-oriented thinking [23]. The following reliability coefficients were obtained in the present study: Total score = .87; difficulty identifying feelings = .87; difficulty describing feelings = .83; externally-oriented thinking = .56.

#### The levels of emotional awareness scale—short version (eLEAS) [12]

The short-version of the LEAS is comprised of 10 scenes, described in two to four sentences, which involve two people (self and other) and are constructed to elicit four types of emotion at five levels of increasing complexity. Individuals read the scenes and then answer questions about how they and another person would feel. Items are given scores of 0 to 4 to indicate the level of emotion for self and other, with higher scores indicating higher levels of emotional complexity. Additionally, a third total score is given, which reflects the higher of the first two scores. A total score of 5 is possible if both self and other receive a score of 4 and the emotions for self and other can be differentiated. Scores for self, other, and total emotional awareness as well as emotional range (the sum of all unique emotion words), multi-level responding (number of items with two or more specific emotion words for self or other), and word count are provided.

#### Facial expression recognition task (FERT) [24]

The FERT is a computerized task of the ability to recognize six basic facial emotions (anger, disgust, fear, happiness, sadness, and surprise) taken from the Pictures of Affect Series [25]. Each emotion was portrayed 10 times at 50% and 10 times at 100% intensity (e.g., small smile, full smile), for a total of 20 presentations of each emotion or 160 stimuli. Each face was presented for 500 ms followed by a blank screen. Participants are asked to choose which emotion was presented from four alternatives presented on the subsequent screen. Individual emotions were collapsed into positive, negative, and total emotional accuracy and response time for positive, negative, and total emotions to increase reliability. Coefficient alphas for positive, negative, and total emotional accuracy were .53, .72, and .71 respectively; coefficient alphas for positive, negative, and total emotion recognition response time were .83, .87, and .92 respectively. Scores on accuracy (greater scores represent higher percent accuracy) and time (higher scores indicate greater lengths of time in ms) are presented.

#### The depression anxiety stress scale—21 (DASS-21) [26]

The DASS-21 is a 21-item measure of depressive, anxiety, and stress-related symptoms experienced over the previous week. Higher scores are indicative of greater endorsement of symptoms of depression, anxiety, and stress. The DASS-21 depression subscale (DASS-21-D) was used to assess negative affect. This subscale has shown evidence of good internal consistency reliability in university samples with alphas ranging from .83–.85 [27, 28]. In the present study, the DASS-21 had an alpha of .92.

#### The Wechsler memory scale, third edition, faces subtest (WMS—faces) [29]

The WMS--Faces includes 24 pictures of human faces. Each face is presented for 2 s in an ordered sequence. Following their administration, participants are presented with a second set of 48 faces comprised of the original 24 faces and 24 novel faces. Participants are asked to indicate whether the face being presented is part of the original group. One point is awarded for each correct response (ranging from 0 to 48). Although standardized scores are available, raw scores were used given the limited age range in this study. The manual reports a reliability coefficient of .77 for ages 18–64. The WMS—Faces was administered to control for individual differences in facial memory skills, as distinct from emotion recognition skills, per recommendations [30].

#### Manipulation check

A manipulation check was used to examine the efficacy of the SFA manipulation. The manipulation check contained 3 items assessing knowledge of being recorded, including: a) “Were you told by the researcher that you were being recorded?” with response options of “yes,” “no,” or “I don’t know”; b) “How certain are you that you were being recorded?” with response options ranging from 1, *not at all*, to 7, *very much*; and c) “How aware were you of the camera recording you during the facial expression task” with response options ranging from 1, *not at all*, to 7, *very much*.

#### Demographics

Participants provided their age, year in school, and racial and ethnic identification. Height and weight were measured at the end of the study.

### Procedures

Participants were recruited from an on-line study registration system. To screen for eligibility, a recruitment statement, the EAT-26, and a few brief demographic questions were administered. As noted before, women who scored greater than or equal to 19 were included in the High-EAT group (*n* = 40). Women who scored at or below 3 were included in the Low-EAT group (*n* = 39).

Eligible participants came to the lab where they provided informed consent and completed the TAS-20 and DASS-21. Next, they were randomly assigned to the SFA or n-SFA conditions. As video recording has been reliably shown to induce self-focus [20], participants in the SFA condition were informed that their participation would be recorded. For the SFA condition, the research assistant (RA) placed a webcam on top of the computer monitor where it was clearly visible and began recording. Participants could see themselves being recorded on the computer monitor before the RA began the study. The control group did not receive these instructions and the webcam was removed. Both groups then received instructions on completing the LEAS and FERT. Finally, the RA administered the WMS-Faces, manipulation check, and demographics form. When complete, height and weight were measured such that participants could not see their measurements. All participants were thanked for their time and given a brief explanation about the purpose of the recording. Participants in the SFA condition were notified that their video would not be used and could watch while the file was deleted. Participants in the High-EAT group were given a handout with on-line and local resources for the treatment of disordered eating.

### Statistical analyses

Measures of frequency, central tendency, and distribution were used to describe the participants and their scores. Differences between eating disorder symptom groups (High-EAT, Low-EAT) on the DASS-21-D and TAS-20 (pre-SFA manipulation) were examined via one-way ANOVAs and ANCOVAs. Scores on the LEAS and FERT (post-SFA manipulation) were examined via two-way ANCOVAs.

## Results

### Manipulation check

The majority of participants (77 of 79) correctly identified whether or not they had been recorded. Two n-SFA participants were unsure if they had been recorded, but if so, responded that they were unaware of it were thus retained for analysis. On a 7-point scale ranging from 1, *not at all*, to 7, *very much*, SFA participants scored *M* = 6.41 (*SD* = 1.20) on certainty of being recorded and *M* = 4.27 (*SD* = 2.15) on awareness of the recording during their performance, suggesting at least a moderate level of self-awareness.

### Descriptive statistics

There were no significant demographic differences among the groups (i.e., age, body mass index, year in school, race, ethnicity). On the DASS-21-D, the High-EAT group scored significantly higher than the Low-EAT group, *F* (1, 77) = 31.06, *p* < .001, *ɳ*_p_^2^ = .287. The correlation between disordered eating group and the DASS-21-D was *r* = .54, *p* < 001. DASS-21-D scores were statistically controlled in subsequent analyses. Although there were no significant differences among the groups on the WMS—Faces, correlations showed that it was significantly correlated with aspects of Facial Emotion Recognition and thus the WMS—Faces was statistically controlled in analysis of the FERT. Correlations among the study variables are available upon request.

### Primary analyses

#### Pre-SFA manipulation

On the TAS-20, The High-EAT group scored higher than the Low-EAT group on the TAS-20 total score, *F* (1, 77) = 22.40, *p* < .001, *ɳ*_p_^2^ = .225, difficulty identifying feelings, *F* (1, 77) = 33.64, *p* < .001, *ɳ*_p_^2^ = .304, and difficulty describing feelings, *F* (1, 77) = 13.40, *p* < .001, *ɳ*_p_^2^ = .148, but not externally oriented thinking, *F* (1, 77) = 1.67, *p* = .200, *ɳ*_p_^2^ = .021 (Supplement Table 1). After controlling for the DASS-21-D, significant effects remained only for the TAS total score, *F* (1, 76) = 4.47, *p* = .038, *ɳ*_p_^2^ = .057 and difficulty identifying feelings, *F* (1, 76) = 8.71, *p* = .004, *ɳ*_p_^2^ = .103. There were no significant differences on the subscales of difficulty describing feelings, *F* (1, 76) = 1.84, *p* = .179, *ɳ*_p_^2^ = .024 or externally oriented thinking, *F* (1, 76) = 0.22, *p* = .644, *ɳ*_p_^2^ = .003 (Supplement Table 2). From another perspective, the High-EAT group contained a greater number of participants meeting criteria for possible (*n* = 9) and probable (*n* = 10) alexithymia than the Low-EAT group (*n* = 3 for possible; *n* = 0 for probable alexithymia), Fisher’s Exact Test, *p* < .001.

#### Post-SFA manipulation

Main effects of eating disorder status were examined first (see Table 1; Supplement Table 2). With regard to the e-LEAS, the High-EAT group scored significantly lower than the Low-EAT group on the total scale, emotional awareness percentile, and self emotional awareness, but not on other emotional awareness. The High-EAT group also had a lower number of multi-level responses and exhibited a trend toward a lower emotional range score. Differences in scores did not appear to be driven by differences in word count. There were no significant main effects of eating disorder status on the FERT.

**Table 1:** Effects of eating disorder symptom group, self-focused attention, and their interaction on the e-LEAS and FERT while controlling for the DASS-21-D

Measure	Eating Group		SFA Group		Interaction	
	*F*	*p*	*F*	*p*	*F*	*p*
e-LEAS						
Total Emotional Awareness	4.59^a^	.036	1.32	.255	0.43	.515
Emotional Awareness Percentile	3.97^a^	.050	0.95	.332	0.22	.643
Emotional Range	3.49	.066	0.01	.943	0.12	.730
Multi-Level Responses	6.41^a^	.013	0.01	.911	0.26	.613
Total Word Count	1.53	.219	0.01	.919	0.17	.685
Self Emotional Awareness	5.17^a^	.026	5.84^b^	.019	0.53	.468
Self Word Count	0.76	.387	0.14	.713	0.24	.626
Other Emotional Awareness	2.49	.119	0.24	.624	0.26	.615
Other Word Count	2.69	.105	0.46	.498	0.08	.773
FERT						
Total % Accurate	0.65	.422	2.08	.153	0.07	.790
Total Response Time (ms)	0.05	.882	1.85	.178	1.23	.271
Negative Emotions % Accurate	0.40	.531	2.17	.145	0.14	.706
Negative Emotions Response Time (ms)	0.14	.706	4.01^b^	.049	0.02	.904
Positive Emotions % Accurate	0.38	.540	1.41	.239	1.20	.278
Positive Emotions Response Time (ms)	0.61	.437	1.71	.196	0.02	.888

*Note*. Eating Group (High-EAT versus Low-EAT); SFA Group (SFA versus n-SFA); e-LEAS = The Levels of Emotional Awareness Scale – Short Version (electronic scoring); FERT = Facial Expression Recognition Task; ^a^High-EAT group scored lower; ^b^SFA group scored lower

Main effects of SFA were then examined (see Table 1; Supplement Table 3). On the e-LEAS, there was a main effect of SFA on self emotional awareness with the SFA group performing more poorly than the n-SFA group. On the FERT, the SFA women more quickly identified negative emotions than the n-SFA women. There were no other main effects on the e-LEAS or FERT.

Interactions between eating disorder status and SFA were examined next. Contrary to hypothesis, there were no statistically significant interactions on the e-LEAS or FERT (see Table 1; Supplement Table 4).

## Discussion

The purpose of the present study was to examine differences between women with high and low disordered eating symptom scores on self-reported alexithymia, emotional awareness, and facial emotion recognition while manipulating SFA using a 2 × 2 experimental design. This is one of a few studies to examine emotional awareness and facial emotion recognition in an “at-risk” sample and the first to test the influence of experimentally induced SFA on emotional awareness and facial emotion recognition among an “at-risk” or any eating disorder sample.

Main effects of disordered eating status were evident on the TAS-20 and e-LEAS. With regard to alexithymia, High-EAT women scored significantly worse than Low-EAT women on difficulty identifying feelings, but scored similarly on difficulty describing feelings and externally-oriented thinking. In contrast, High-EAT women evidenced lower total, self, and other emotional awareness scores on the e-LEAS, an instrument designed to measure the ability to describe feelings. Further, High-EAT women provided fewer multi-level responses and a shorter emotional range, supporting the idea that screening and perhaps subclinical samples evidence the same deficits as clinical samples [5].

Consistent with previous research, differences between eating disorder groups and healthy controls are often attenuated after controlling for negative affect on the TAS-20, but remain apparent on the LEAS [6–8]. One study [14] found no significant differences between women high and low in eating disorder symptoms on the TAS-20 after controlling for depression or anxiety, but also found no significant differences on the LEAS, leading them to conclude that although women with anorexia nervosa believe they are worse at describing emotions, they perform similarly to healthy controls. They suggest that the LEAS is superior to the TAS-20 as it assesses the actual ability to describe emotions versus beliefs about this ability. Others have speculated that the LEAS and TAS-20 measure different constructs [8]. Indeed, there are some differences in definition, with the TAS-20 purporting to measure the ability to recognize and describe one’s own emotions and the LEAS measuring the ability to describe emotions in both oneself and others. Thus, these two aspects of theory of mind appear may be distinct.

Further, there were no main effects of disordered eating status on facial emotion recognition. Previous research has shown that these deficits tend to be driven by samples diagnosed with anorexia nervosa [2, 3, 10] whereas our sample was comprised of a screening sample. Further, the FERT was a forced-choice emotion recognition task and as such may not have been as sensitive to differences between women scoring high and low on a measure of eating disorder symptoms as free naming tasks or tasks depicting mixed emotional states. Alternatively, an intact ability to identify emotional expressions in others may characterize a screening or “at-risk” sample versus a clinical eating disorder sample.

This is also one of the few studies to examine the role of self-focused attention on emotion recognition. There was a main effect for SFA with women in the SFA group earning lower self emotional awareness scores, suggesting that SFA may have detracted from the ability to describe one’s own feelings. It is interesting that SFA was associated with lower self but not other emotional awareness as was found in a previous study [10]. One might think that greater SFA would be associated with greater attention to one’s emotions. However, the task involved describing how they and someone else would feel in a given scenario, not how they were presently feeling. Thus, increased focus on internal states, such as how they were presently feeling, might interfere with the ability to consider how they would feel in another scenario. Also unknown is the content of SFA, specifically whether it was self-relevant (e.g., “I’m bad at this”) or non-self-relevant (“This is a strange task”). Decreased attention to one’s own emotional state, consistent with non-self-relevant SFA, may have been less distressing and/or more reinforcing.

There was also a main effect of SFA on negative emotion response time with women in the SFA condition being quicker to identify negative emotions. While SFA is associated with multiple forms of psychopathology [15], it has a strong association with negative affect across observational studies (*d* = .51) and experimental studies (*d* = .44) [16]. Further, while the meta-construct model [15] conceptualizes SFA as a general vulnerability for various forms of psychopathology, other models conceptualize SFA as a risk factor specific to disorders of negative affect [31]. Indeed, SFA was associated with symptoms of depression among patients with anorexia nervosa during recall of a negative autobiographical memory [17].

It should also be noted that these results were limited in scope and did not apply to accuracy in identifying emotions. According to the meta-construct model [15], as SFA increases, the ability to attend to external events decreases. However, increased SFA is not the same as excessive SFA; thus, participants may not have reached a level at which their ability to attend to external events was sufficiently compromised or consistently compromised [15]. Future studies would benefit from inclusion of brief measures of self-awareness both before and after experimental manipulations.

Finally, the present study sought to determine whether women with disordered eating symptoms would be more susceptible to a manipulation that increased SFA. Contrary to expectations, no significant interaction between eating disorder status and SFA was found. It is difficult to interpret whether symptomatic women experienced a greater increase in SFA than asymptomatic women given the present study design. According to the meta-construct model [15], symptomatic women should have experienced a greater increase in SFA than asymptomatic women. However, the indicator of this increase was performance on a facial emotion recognition task and not specifically SFA. Further, one should be mindful that this was a sample that screened positive for eating disorder symptoms (i.e., not a treatment sample) that may have been less susceptible to these effects. It is also possible that the task itself distracted women from SFA. That is, instead of evaluating their performance, participants may have evaluated the task itself. Indeed, it has been noted that experimental manipulations may divert attention toward external processes such as the tasks involved [15]. Another possibility is that the tasks were not relevant to disordered eating. Perhaps an experiment involving aspects of physical appearance would be more appropriate. Finally, the SFA manipulation in our study may not have been of sufficient intensity or duration to produce the desired effect. Further research should investigate the associations between self-report and experimentally induced SFA and aspects of disordered eating as well as the relationship between SFA, social comparison, and social evaluative anxiety.

### Implications

The results of this study have several implications. First, they illustrate that difficulties describing one’s own and others’ emotions, but not facial emotion recognition, are present among samples that screen positive for disordered eating, suggesting that these difficulties occur across the spectrum of disordered eating and are not limited to treatment samples. Second, these results confirm earlier observations regarding the role of negative affect in self-reported alexithymia. Using a performance-based task, such as the e-LEAS, and measuring negative affect is indicated. Third, it may be beneficial to assess emotional awareness and/or the tendency to avoid negative emotions among women presenting with disordered eating. Interventions to improve self and other emotional awareness and/or that increase emotional tolerance (e.g., dialectical behavior therapy) [32] may be indicated among women showing impairments in these areas. Finally, consistent with previous literature [15, 16], results suggest that SFA, regardless of eating disorder status, may prime participants toward negative emotion recognition. Therefore, interventions to decrease SFA, such as attentional training therapy [33] may decrease this emotional vulnerability.

### Limitations

The present study was comprised of an all-female screening sample from a private undergraduate university. Despite adequate representation of female undergraduates with and without eating disorder symptoms, these results may not generalize well to a clinical population. Further, the manipulation check had a detectable, albeit moderate effect. Efforts to increase the effectiveness of this manipulation should be made. Additionally, we did not adjust for multiple tests given the apriori hypotheses; however, this practice may have inflated the type I error rate. Finally, the utilization of a 2 × 2 design and the splitting into four groups resulted in a somewhat small sample (i.e., *n* = 20 per group). Further research with a larger sample size is warranted.

## Conclusions

Continued examination of emotional recognition in oneself and others may help inform observations of interpersonal difficulties among persons with disordered eating.

## Supplementary information

**Additional file 1.** Supplement Table 1. Main effect comparisons between disordered eating groups (anovas). Supplement Table 2. Main effect comparisons between disordered eating groups controlling for depression. Supplement Table 3. Main effect comparisons between SFA and n-SFA groups controlling for depression. Supplement Table 4. Interactions between disordered eating group and self-focused attention controlling for depression.

## Data Availability

Data and materials analyzed during this study are available from the corresponding author upon reasonable request. The data are not publicly available as the data are part of a larger dataset currently being analyzed.

## Abbreviations

ANCOVA: Analysis of covariance
ANOVA: Analysis of variance
DASS-21: Depression Anxiety Stress Scale – 21
DASS-21-D: Depression Anxiety Stress Scale—21 Depression Subscale
e-LEAS: Electronic Levels of Emotional Awareness Scale
EAT-26: Eating Attitudes Test--26
FERT: Facial Expression Recognition Task
High-EAT: High scores (> 19) on the EAT-26
Low-EAT: Low scores (< 3) on the EAT-26
M: Mean
n-SFA: Non-self-focused attention
SD: Standard deviation
SE: Standard error
SFA: Self-focused attention
TAS-20: Toronto Alexithymia Scale
WMS: Wechsler Memory Scale
