# Supplementary material for: Differences in alexithymia, emotional awareness, and facial emotion recognition under conditions of self-focused attention among women with high and low eating disorder symptoms: a 2 x 2 experimental study
Source: J Eat Disord. 2020 Jun 22;8:28. doi: 10.1186/s40337-020-00304-5 (PMC7309995; doi:10.1186/s40337-020-00304-5)
Supplement: Supplementary file 1 — Additional file 1. Supplement Table 1. Main effect comparisons between disordered eating groups (anovas). Supplement Table 2. Main effect comparisons between disordered eating groups controlling for depression. Supplement Table 3. Main effect comparisons between SFA and n-SFA groups controlling for depression. Supplement Table 4. Interactions between disordered eating group and self-focused attention controlling for depression. [file 40337_2020_304_MOESM1_ESM.docx]

Supplement Table 1. Main effect comparisons between disordered eating groups (anovas).

| Measure | | Low-EAT | | High-EAT | |  | |  | |  | |  |
| --- | --- | --- | --- | --- | --- | --- | --- | --- | --- | --- | --- | --- |
|  | | *M* | *SD* | *M* | *SD* | | *F* | | *p* | | *ɳ*_p_^2^ | |
| TAS | |  |  |  |  | |  | |  | |  | |
|  | Total Score | 41.59 | 6.73 | 51.53 | 11.30 | | 22.40 | | .000 | | .225 | |
|  | Identify Feelings | 12.15 | 3.08 | 17.78 | 5.23 | | 33.64 | | .000 | | .304 | |
|  | Describe Feelings | 10.97 | 3.34 | 14.33 | 4.67 | | 13.40 | | .000 | | .148 | |
|  | External Thinking | 18.46 | 2.70 | 19.43 | 3.81 | | 1.67 | | .200 | | .021 | |
| DASS-21 | |  |  |  |  | |  | |  | |  | |
|  | Depression Subscale | 8.59 | 2.16 | 13.60 | 5.19 | | 31.06 | | .000 | | .287 | |

*Note*. Low-EAT = low eating disorder symptom score group; High-EAT = high eating disorder symptom score group; TAS = Toronto Alexithymia Scale – 20; e-LEAS = The Levels of Emotional Awareness Scale – Short Version; FERT = Facial Expression Recognition Task

Supplement Table 2. Main effect comparisons between disordered eating groups controlling for depression.

| Measure | | Low-EAT | | High-EAT | |  | |  | |  | |  |
| --- | --- | --- | --- | --- | --- | --- | --- | --- | --- | --- | --- | --- |
|  | | *M* | *SE* | *M* | *SE* | | *F* | | *p* | | *ɳ*_p_^2^ | |
| TAS^a^ | |  |  |  |  | |  | |  | |  | |
|  | Total Score | 44.22 | 1.49 | 48.96 | 1.47 | | 4.47 | | .038 | | .056 | |
|  | Identify Feelings | 13.50 | 0.66 | 16.47 | 0.66 | | 8.71 | | .004 | | .103 | |
|  | Describe Feelings | 11.98 | 0.66 | 13.34 | 0.65 | | 1.84 | | .179 | | .024 | |
|  | External Thinking | 18.74 | 0.58 | 19.15 | 0.57 | | 0.22 | | .644 | | .003 | |
| eLEAS^b^ | |  |  |  |  | |  | |  | |  | |
|  | Total Emotional Awareness | 37.92 | 0.69 | 35.69 | 0.68 | | 4.59 | | .036 | | .058 | |
|  | Emotional Awareness Percentile | 66.88 | 4.74 | 52.55 | 4.68 | | 3.97 | | .050 | | .051 | |
|  | Emotional Range | 74.14 | 3.09 | 65.38 | 3.05 | | 3.49 | | .066 | | .045 | |
|  | Multi-Level Responses | 6.46 | 0.39 | 4.97 | 0.38 | | 6.41 | | .013 | | .080 | |
|  | Total Word Count | 321.24 | 25.67 | 272.99 | 25.31 | | 1.53 | | .219 | | .020 | |
|  | Self Emotional Awareness | 33.22 | 0.63 | 31.07 | 0.61 | | 5.17 | | .026 | | .075 | |
|  | Self Word Count | 162.33 | 14.45 | 143.23 | 14.25 | | 0.76 | | .387 | | .010 | |
|  | Other Emotional Awareness | 30.71 | 0.75 | 28.92 | 0.74 | | 2.49 | | .119 | | .033 | |
|  | Other Word Count | 158.91 | 11.71 | 129.76 | 11.55 | | 2.69 | | .105 | | .035 | |
| FERT^b^ | |  |  |  |  | |  | |  | |  | |
|  | Total % Accurate | 67.62 | 1.13 | 69.01 | 1.11 | | 0.65 | | .422 | | .01 | |
|  | Total Response Time (ms) | 1477.99 | 84.18 | 1506.53 | 82.00 | | 0.05 | | .822 | | .001 | |
|  | Negative Emotions % Accurate | 61.33 | 1,63 | 62.88 | 1.60 | | 0.40 | | .531 | | .005 | |
|  | Negative Emotions Response Time (ms) | 1517.88 | 88.87 | 1568.92 | 87.61 | | 0.14 | | .706 | | .002 | |
|  | Positive Emotions % Accurate | 80.66 | 1.47 | 82.03 | 1.45 | | 0.38 | | .540 | | .005 | |
|  | Positive Emotions Response Time (ms) | 1461.17 | 108.54 | 1589.93 | 107.00 | | 0.61 | | .437 | | .008 | |

*Note*. ^a^analyzed with one-way ANCOVAs; ^b^analyzed with 2 x 2 ANCOVAs; Low-EAT = low eating disorder symptom score group; High-EAT = high eating disorder symptom score group; TAS = Toronto Alexithymia Scale – 20; e-LEAS = The Levels of Emotional Awareness Scale – Short Version; FERT = Facial Expression Recognition Task.

Supplement Table 3. Main effect comparisons between SFA and n-SFA groups controlling for depression.

| Measure | | SFA | | n-SFA | |  |  | |  | |
| --- | --- | --- | --- | --- | --- | --- | --- | --- | --- | --- |
|  | | *M* | *SD* | *M* | *SD* | *F* | | *p* | | *ɳ*_p_^2^ |
| eLEAS^a^ | |  |  |  |  |  | |  | |  |
|  | Total Emotional Awareness | 36.30 | 0.63 | 37.31 | 0.62 | 1.32 | | .255 | | .017 |
|  | Emotional Awareness Percentile | 56.75 | 4.32 | 62.68 | 4.27 | 0.95 | | .332 | | .013 |
|  | Emotional Range | 69.90 | 2.82 | 69.62 | 2.78 | 0.01 | | .943 | | .000 |
|  | Multi-Level Responses | 5.69 | 0.35 | 5.74 | 0.35 | 0.01 | | .911 | | .000 |
|  | Total Word Count | 298.80 | 23.41 | 295.43 | 23.11 | 0.17 | | .685 | | .002 |
|  | Self Emotional Awareness | 31.18 | 0.54 | 33.12 | 0.69 | 5.84 | | .019 | | .084 |
|  | Self Word Count | 149.35 | 13.18 | 156.21 | 13.01 | 0.14 | | .713 | | .002 |
|  | Other Emotional Awareness | 29.58 | 0.69 | 30.05 | 0.68 | 0.24 | | .624 | | .003 |
|  | Other Word Count | 149.45 | 10.68 | 139.22 | 10.55 | 0.46 | | .498 | | .006 |
| FERT^a^ | |  |  |  |  |  | |  | |  |
|  | Total % Accurate | 67.26 | 1.03 | 69.37 | 1.02 | 2.08 | | .153 | | .028 |
|  | Total Response Time (ms) | 1418.77 | 76.30 | 1565.76 | 75.34 | 1.85 | | .178 | | .025 |
|  | Negative Emotions % Accurate | 60.55 | 1.49 | 63.66 | 1.47 | 2.17 | | .145 | | .029 |
|  | Negative Emotions Response Time (ms) | 1427.80 | 81.52 | 1658.99 | 80.49 | 4.01 | | .049 | | .052 |
|  | Positive Emotions % Accurate | 82.48 | 1.35 | 80.22 | 1.33 | 1.41 | | .239 | | .019 |
|  | Positive Emotions Response Time (ms) | 1433.50 | 99.57 | 1617.60 | 98.31 | 1.71 | | .196 | | .023 |

*Note*. ^a^analyzed with 2 x 2 ANCOVAs; n-SFA = non-self-focused attention group; SFA = self-focused attention group; e-LEAS = The Levels of Emotional Awareness Scale – Short Version; FERT = Facial Expression Recognition Task.

Supplement Table 4. Interactions between disordered eating group and self-focused attention controlling for depression.

| Measure | | Low-EAT | | | | High-EAT | | | |  |  |  |
| --- | --- | --- | --- | --- | --- | --- | --- | --- | --- | --- | --- | --- |
|  | | *SFA* | | *n-SFA* | | *SFA* | | *n-SFA* | |  |  |  |
|  | | *M* | *SE* | *M* | *SE* | *M* | *SE* | *M* | *SE* | *F* | *p* | *ɳ*_p_^2^ |
| eLEAS^a^ | |  |  |  |  |  |  |  |  |  |  |  |
|  | Total Emotional Awareness | 37.13 | 0.93 | 38.71 | 0.93 | 35.47 | 0.92 | 35.90 | 0.92 | 0.43 | .515 | .006 |
|  | Emotional Awareness Percentile | 62.50 | 6.41 | 71.27 | 6.44 | 51.00 | 6.32 | 54.10 | 6.34 | 0.22 | .643 | .003 |
|  | Emotional Range | 73.59 | 4.17 | 74.69 | 4.20 | 66.21 | 4.12 | 64.55 | 4.13 | 0.12 | .730 | .002 |
|  | Multi-Level Responses | 6.31 | 0.52 | 6.62 | 0.53 | 5.07 | 0.52 | 4.87 | 0.52 | 0.26 | .613 | .003 |
|  | Total Word Count | 316.21 | 34.67 | 326.26 | 34.88 | 281.39 | 34.20 | 264.59 | 34.33 | 0.17 | .685 | .002 |
|  | Self Emotional Awareness | 31.96 | 0.79 | 34.49 | 0.91 | 30.40 | 0.80 | 31.75 | 0.87 | 0.53 | .468 | .008 |
|  | Self Word Count | 254.37 | 19.52 | 170.29 | 19.64 | 144.34 | 19.25 | 142.12 | 19.32 | 0.24 | .626 | .003 |
|  | Other Emotional Awareness | 30.23 | 1.01 | 31.19 | 1.02 | 28.92 | 1.00 | 28.91 | 1.00 | 0.26 | .615 | .003 |
|  | Other Word Count | 161.85 | 15.82 | 155.98 | 15.92 | 137.05 | 15.61 | 122.47 | 15.66 | 0.08 | .773 | .001 |
| FERT^a^ | |  |  |  |  |  |  |  |  |  |  |  |
|  | Total % Accurate | 66.76 | 1.52 | 68.49 | 1.54 | 67.75 | 1.51 | 70.25 | 1.50 | 0.07 | .790 | .001 |
|  | Total Response Time (ms) | 1345.34 | 112.35 | 1610.64 | 113.64 | 1492.19 | 111.43 | 1520.88 | 111.21 | 1.23 | .271 | .017 |
|  | Negative Emotions % Accurate | 59.38 | 2.20 | 63.27 | 2.22 | 61.72 | 2.17 | 64.04 | 2.17 | 0.14 | .706 | .002 |
|  | Negative Emotions Response Time (ms) | 1409.17 | 120.04 | 1626.59 | 121.41 | 1446.43 | 119.05 | 1681.40 | 118.82 | 0.02 | .904 | .000 |
|  | Positive Emotions % Accurate | 82.82 | 1.98 | 78.50 | 2.00 | 82.14 | 1.96 | 81.93 | 1.96 | 1.20 | .278 | .016 |
|  | Positive Emotions Response Time (ms) | 1359.30 | 146.61 | 1563.05 | 148.28 | 1507.71 | 145.40 | 1672.15 | 145.12 | 0.02 | .888 | .000 |

*Note*. ^a^analyzed with 2 x 2 ANCOVAs; Low-EAT = low eating disorder symptom score group; High-EAT = high eating disorder symptom score group; n-SFA = non-self-focused attention group; SFA = self-focused attention group; e-LEAS = The Levels of Emotional Awareness Scale – Short Version; FERT = Facial Expression Recognition Task.
